# Advanced In Situ TEM Microchip with Excellent Temperature Uniformity and High Spatial Resolution

**DOI:** 10.3390/s23094470

**Published:** 2023-05-04

**Authors:** Xuelin Zhang, Yufan Zhou, Ying Chen, Ming Li, Haitao Yu, Xinxin Li

**Affiliations:** 1 State Key Laboratory of Transducer Technology, Shanghai Institute of Microsystem and Information Technology, Chinese Academy of Sciences, Shanghai 200050, China; 2 School of Microelectronics, University of Chinese Academy of Sciences, Beijing 100049, China

**Keywords:** MEMS, in situ TEM, microchip, gas cell, temperature uniformity

## Abstract

Transmission electron microscopy (TEM) is a highly effective method for scientific research, providing comprehensive analysis and characterization. However, traditional TEM is limited to observing static material structures at room temperature within a high-vacuum environment. To address this limitation, a microchip was developed for in situ TEM characterization, enabling the real-time study of material structure evolution and chemical process mechanisms. This microchip, based on microelectromechanical System (MEMS) technology, is capable of introducing multi-physics stimulation and can be used in conjunction with TEM to investigate the dynamic changes of matter in gas and high-temperature environments. The microchip design ensures a high-temperature uniformity in the sample observation area, and a system of tests was established to verify its performance. Results show that the temperature uniformity of 10 real-time observation windows with a total area of up to 1130 μm^2^ exceeded 95%, and the spatial resolution reached the lattice level, even in a flowing atmosphere of 1 bar.

## 1. Introduction

TEM has proven to be an extremely effective tool for exploring the microscopic world. Over the years, impressive advancements in various areas such as field-emission electron guns, aberration correctors, cameras, imaging algorithms, and high-efficiency spectrometers have led to significant improvements in TEM observations [1,2,3]. Today’s TEMs are capable of achieving sub-angstrom levels of spatial resolution and several electron millivolts of energy resolution, providing scientists with a unique perspective for analysis [4,5]. With these advanced capabilities, TEM has become an indispensable tool in various fields such as material science, nanotechnology, and biology.

Traditional TEM is limited to studying the static morphology and structure of a sample, which is known as “ex situ” observation [6,7,8]. This approach may miss important dynamic events that occur during chemical processes [9,10,11,12,13,14]. It is crucial to record chemical reactions in real time to fully understand the mechanism of atomic-level structural evolution [15,16,17]. This is where in situ TEM comes in, which involves subjecting the sample to one or multiple physical stimuli, such as heat, light, force, cooling, pressure, gases, and liquids, inside the TEM column to observe the real-time evolution of the material. In situ TEM has been used to study various materials, including crystalline materials [18,19], energy storage and conversion [1,20], catalytic nanomaterials [21,22,23,24,25], and biomedical research [4,26]. Therefore, the development of such advanced in situ TEM is crucial for the progress of materials science and engineering, and for advancing our understanding of the world.

An approach for importing gases such as H_2_, H_2_S, O_2_, or mixtures into a TEM is known as environmental TEM (ETEM). The height of gas in the electron column injected by ETEM defines the length of transmission electron passing through it. However, this means that the path of electrons is short and the gas pressure must be sacrificed to guarantee resolution. Typically, ETEM achieves a gas pressure of 10 mbar [27]. There are various ways to introduce heated stimuli. One method involves using a furnace-type holder to heat the entire TEM grid to 1000 °C, which results in serious thermal drift [28]. Another way is to use a tungsten wire to introduce a heated field, which raises the temperature to 1500 °C. In this approach, the powder sample is coated on the surface of the tungsten wire [29]. However, the drawback is that the visible area of samples is limited.

By combining MEMS technology with in situ analysis technology, a highly stable and uniform micro-reactor has been developed for real-time observation of samples injected into a heated gaseous atmosphere. However, these gas cell chips have the disadvantage of poor uniformity in heating temperature. Héctor Hugo Pérez-Garza utilized MEMS technology to create a gas cell with a uniform heating wire line width and a window area of less than 850 μm^2^ [30]. Vendelbo measured a maximum temperature difference of 56 °C using electron energy loss spectroscopy [31]. Mele reported variations in temperature uniformity in the window region from 10% to 15% [32].

In this study, we designed and fabricated an MEMS chip that can be used with a sample holder to create a controlled gas and heated environment in a TEM for in situ sample observation. Through the optimization of the heating filament design, the observation window of the microchip achieved a high level of temperature uniformity, with over 95% uniformity across a total visible area of up to 1130 μm^2^. With this microchip, we were able to achieve lattice-level resolution during in situ observation of palladium particles in both stationary and flowing atmospheres. By leveraging the advanced features of the microchip, scientists can gain a deeper understanding of various materials and their behavior, leading to groundbreaking discoveries and innovations in various fields.

## 2. Design

### 2.1. Microchip Configuration

**Figure 1:**
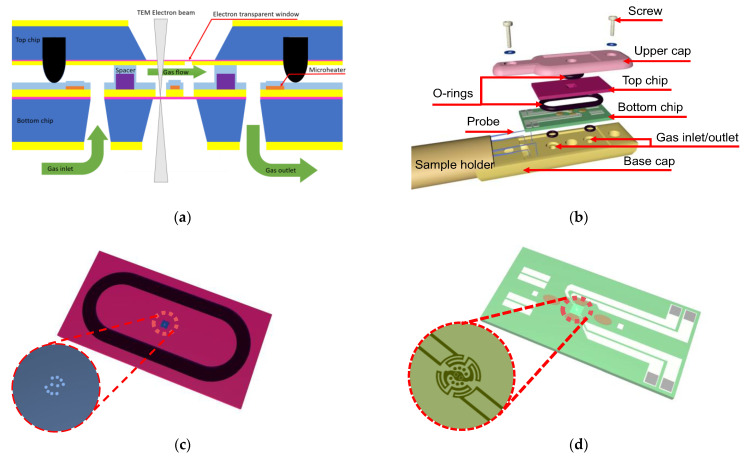
Structural diagrams of the microchip: (**a**) cross-sectional view of the microchip and O-rings after assembly; (**b**) schematic diagram of the assembly with the sample holder; (**c**) top chip; (**d**) bottom chip.

A cross-sectional view of the novel in situ TEM microchip proposed to overcome the limitations of traditional TEM is shown in Figure 1a. The microchip consists of two highly advanced MEMS chips, each with a unique set of features. The bottom chip boasts an electron transmission window, metal wires for heating, and through-holes for introducing gas, making it an ideal platform for in situ experiments. Meanwhile, the top chip has a series of electron transmission windows and a deep O-groove that further enhances the device’s sealing performance. When the two chips are assembled, they form a sealed microreactor that includes an inlet and outlet. The microreactor can provide a precise temperature field for the sample while simultaneously introducing reaction gases, allowing researchers to conduct highly accurate and controlled experiments. This novel design offers significant advantages over traditional TEM, enabling researchers to observe the reaction process in real time and obtain insights into the dynamics of the reaction.

To utilize the microchip, researchers must carefully assemble it with a sample holder composed of two parts and place it within the column of a TEM. The upper and base portions of the sample holder feature electron beam through holes that are precisely aligned with the large overhanging membrane of the top and bottom chips, enabling researchers to conduct experiments with unparalleled accuracy. To ensure an airtight gas environment, the upper cap of the sample holder, the base cap, screws, and O-rings work together in perfect harmony. The lower part of the sample holder connects to the gas inlet and outlet of the microchip, enabling researchers to introduce gas and control the reaction process. Additionally, four probes on the sample holder connect to the four pads of the lower chip, enabling scientists to introduce current for heating and measurement. The assembly diagram of the microchip, O-ring, and sample holder is depicted in Figure 1b.

### 2.2. Transparent Membrane

To ensure stable and consistent transmission electron beam imaging, it is important to keep the observed particles in a fixed position on the electron transmission window. However, when conducting observations under heated reaction conditions, the electron transmission window and the supporting film may expand thermally and move along with the observed sample. This can cause thermal drift of the sample and even result in the sample moving out of the imaging range. Therefore, it is imperative to choose appropriate materials that can withstand harsh conditions and provide accurate results when conducting transmission electron beam imaging experiments. To prevent this from happening, materials with a small coefficient of thermal expansion that are compatible with MEMS manufacturing technology, such as amorphous silicon nitride, are used [33,34]. By employing such materials, researchers can accurately observe the sample’s behavior under various conditions, allowing for a more in-depth understanding of the material’s properties and reactions.

In addition, when observing samples that are very small, such as nanoparticles with a diameter of only a few tens of nanometers, achieving accurate transfer of the sample to the observation hole can be a daunting task. The microscopic undulations of the window position, combined with the sample’s minuscule size, can lead to incorrect sample placement, thus compromising the experiment’s accuracy. To overcome this challenge, scientists often opt to increase the size of the observation hole as much as possible while ensuring air pressure safety and temperature uniformity. Therefore, it is crucial to employ sophisticated techniques to address the challenge of transferring small samples accurately and consistently while ensuring that the experiments remain safe and reliable.

For instance, electron transmission holes with a diameter of 12 μm and a total observation area of 1130 μm^2^ were designed to maximize the probability of accurate sample placement. Moreover, to minimize interference from the heating wires, the location of outer 8 windows was situated at a considerable distance from the central undulant heated wires. By implementing such precision engineering, scientists can increase the accuracy of their observations and gain new insights into the properties and behavior of nanoparticles, enabling them to develop innovative solutions in various fields.

### 2.3. Temperature Optimization

**Figure 2:**
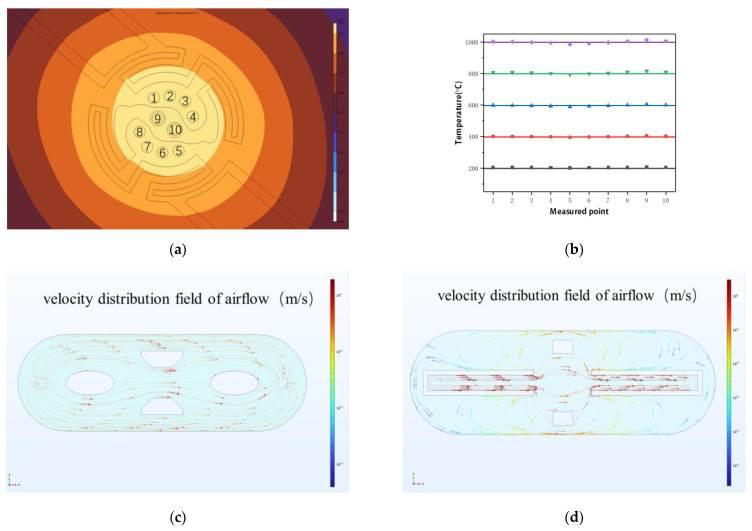

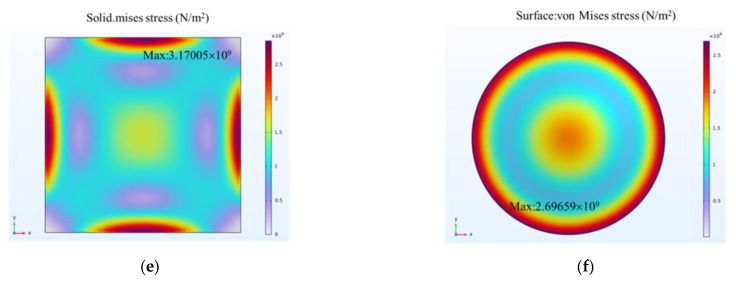
Finite element analysis: (**a**) temperature distribution of the microheater including the locations of 10 windows; (**b**) temperature of 10 windows at 200 °C, 400 °C, 600 °C, 800 °C, and 1000 °C; (**c**) airflow velocity distribution of pin fin design; (**d**) airflow velocity distribution of horseshoe shape spacer design; (**e**) stress simulation of the rectangular supporting membrane with a thickness of 700 nm and a pressure difference of 1 bar on the membrane; (**f**) stress simulation of the circular transparent membrane with a thickness of 30 nm and a pressure difference of 1 bar on the membrane.

Before the heating experiment, the samples were randomly distributed across each observation window. To ensure that chemical reactions occur synchronously on each window, it is crucial to maintain a uniform temperature across all windows. Achieving high temperatures and excellent temperature uniformity in the window area requires effective heat transfer and dissipation mechanisms, which depend on the geometry of the heating element and the choice of materials. The microheater was designed with a meandering and spiraling shape, and the width of the heating line gradually increase from the edge to the center of the heater. This combination of wire heating and heat conduction helps to achieve superior temperature uniformity across the entire window area. Finite element analysis (FEA) indicated that the temperature uniformity across 10 observation windows exceeded 98% at temperatures ranging from 200 °C to 1000 °C (Figure 2b).

Several heating materials are compatible with MEMS processes, but selecting the right material can be challenging. For instance, some nonmetallic materials such as polysilicon have a nonlinear temperature coefficient of resistance (TCR), and most of them have a low electron density and slow temperature response. Tungsten is also not a good option since it is oxidized at 400 °C and volatilized at 800 °C. Platinum has a low melting point, making it unsuitable for high-temperature regions. Therefore, it is crucial to select a material with a high melting point and no high-temperature phase changes. Molybdenum is a good option for a heater material in MEMS processes due to its excellent material performance. It has high electron density, a linear TCR, and a high melting point [35,36]. Additionally, the heater temperature of molybdenum can be precisely controlled using a closed-loop feedback system of four probes. Two pads provide a heating current, while the other two pads measure resistance [30,37]. Overall, molybdenum is a suitable heater material for MEMS processes due to its desirable material properties and precise temperature control.

### 2.4. Airflow and Pressure

The optimization design of gas distribution in microchips has not received much attention in the past. However, with the need for better performance and accuracy, it is becoming increasingly clear that airflow uniformity is a crucial factor that demands optimization. Rapid replacement of residual gas after each gas–solid reaction is of utmost importance, as it can lead to uncertainty and errors in subsequent experimental results. To eliminate the risk of experimental interference from static gas or slow airflow, two spacer designs were analyzed for their airflow velocity distribution fields, as depicted in Figure 2c,d.

The design featuring pin fins (Figure 2c) was ultimately adopted, as it produced an extremely uniform airflow distribution within the microchip. The velocity distribution fields were significantly improved, greatly enhancing the displacement speed of the participating gases. This design innovation ensures that residual gas is effectively removed, avoiding potential errors or uncertainties in the experimental results. With this optimized microchip design, researchers can have greater confidence in their experimental outcomes and focus their efforts on advancing their research.

The window used for TEM observation in gas-phase in situ TEM chips plays a crucial role in ensuring high-quality imaging of nanomaterials at high magnification. This delicate element must meet two key criteria. Firstly, it must possess a high transmittance to the electron beam to produce clear and accurate imaging. As such, the window’s transmitted film should be as thin as possible. Secondly, the window must be able to withstand atmospheric pressure differences, ensuring a high-vacuum environment within the transmission electron microscopy. To achieve this, a relatively thick, large suspension film was used to provide the window with strength. Only in the central part of the suspension film was an extremely thin single layer of silicon nitride film added, which served as the transmission channel for electrons.

During use, the pressure difference between the inside and outside of the in situ TEM chip causes the large suspension film and the transmission window to bend and deform, resulting in deflection much greater than the thickness of the suspension film and window. To address this, FEA was employed to study the stress and deformation levels of the suspension film and transmission window under one atmosphere of pressure (Figure 2e,f). The simulation revealed that the maximum stress generated on the suspension film and transmission window still fell below the fracture stress of silicon nitride [38], assuring their durability and reliability. In short, the development of a strong and transparent observation window is crucial for an effective gas-phase in situ TEM, and the use of advanced simulation techniques is key to ensuring their success.

## 3. Fabrication of the Microchip

**Figure 3:**
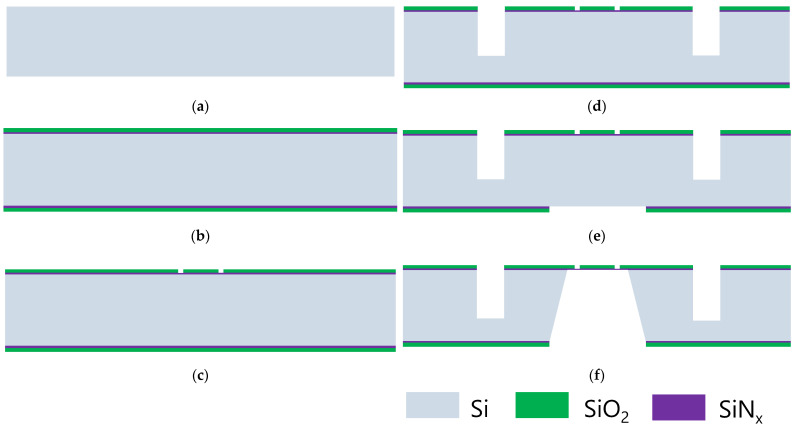
The fabrication process of the top chip: (**a**) double-sided polished wafer; (**b**) deposition of composite film on the wafer; (**c**) definition of the location and size of electron transmission window; (**d**) definition of the O-groove; (**e**) opening the window of wet etching; (**f**) pattern suspended membrane.

**Figure 4:**
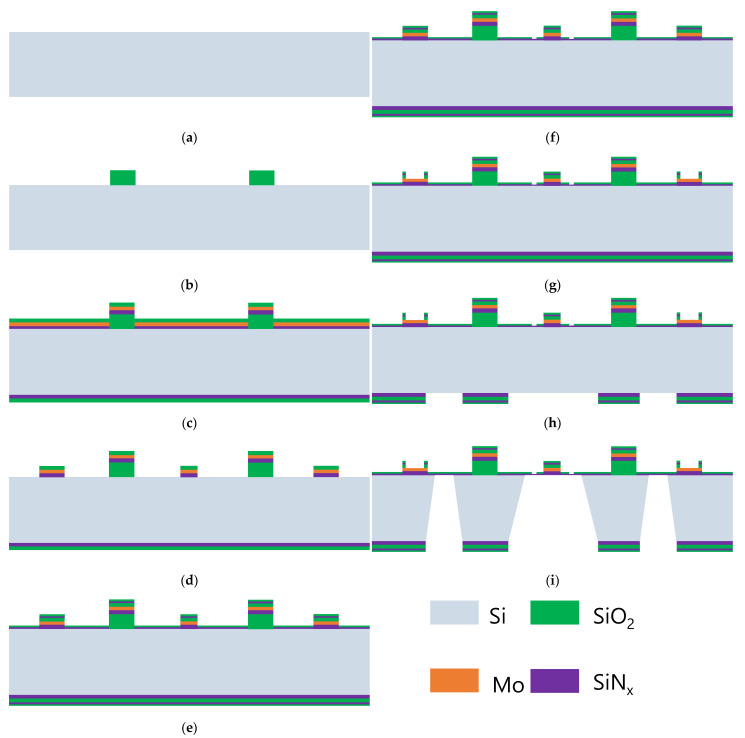
The fabrication process of the bottom chip: (**a**) double-sided polished wafer; (**b**) definition of the spacers on the front side of the wafer; (**c**,**d**) patterning of the metal heater; (**e**) deposition of a composite film on the wafer; (**f**) definition of the location and size of electron transmission window; (**g**) patterning of electrodes; (**h**) opening of the window for wet etching; (**i**) pattern suspended membrane.

Figure 3 and Figure 4 show the fabrication steps for our proposed TEM microchip, including a top chip and a bottom chip in an ordinary non-SOI (100) silicon wafer, the core of which involves constructing a suspended membrane with some transition windows. Firstly, the composite layer on the top of the wafer except for the thinnest SiNx membrane used to define the transition windows of the electron beam must be etched clean. Secondly, the width and height of heating wires should be strictly prepared to ensure that the designed uniform heating function can be achieved accurately. Lastly, the corrosion depth of potassium hydroxide (KOH), h, the width, $w_{0}$, of the corrosion windows on the backside of the wafer, and the width of the supporting rectangle membrane,$w_{1}$, should satisfy the relationship $w_{1}=w_{0}-\sqrt{2}h$. Processes for the top chip and bottom chip all take place on the (100) silicon wafer according to the abovementioned design guidance, as illustrated and shown in Figure 3 and Figure 4.

The fabrication of the top chip is described below.

1. A composite film featuring a SiNx layer and a SiO_2_ layer was deposited on a double-side polished wafer with a thickness of 420 μm. Then, the first photolithography was performed to define the locations and forms of the transition windows of the top chip. Next, buffered oxide etchant (BOE) was used to etch the SiO_2_ with the photoresist as an etching mask. After the corrosion of BOE, a thin layer of amorphous silicon nitride, 30 nm in thickness, was fabricated in the position of the windows to serve as an electron transmission film.
2. To ensure the microreactor’s tightness after its connection with the O-ring, a deep O-groove was fabricated using photolithography and deep reactive ion etching (DRIE). Thereafter, the second photolithography was conducted to define the O-groove width of 700 nm, and silicon deep reactive ion etching was used to a trench depth of 300 μm. After the formation of the O-groove, the photoresist was stripped.
3. After the third photolithography, the length of the corrosion windows was conducted. Next, reactive ion etching (RIE) was used to open the window of wet etching on the backside of the wafer. Then, the front-protected wafer immersed in 30 wt.% 50 °C KOH was corroded from the backside until silicon nitride on the front side. Thereafter, a deep hole was etched beneath the central suspended membrane, which also served as a transmission beam channel.

The fabrication of the bottom chip is described below.

1. After the double-side polished wafer was prepared, a 2 μm thick layer of SiO_2_ was deposited on both sides of the wafer using a furnace, and the film on the top of the wafer on the front side was used to create spacers between the top and bottom chips. Thereafter, the first photolithography was conducted on the front side of the wafer to pattern the spacer shape, which deeply affected the distribution of airflow. The height of the spacers defined by RIE and BOE also had an effect on the airstream in the chamber. Furthermore, the SiO_2_ layer on the backside was etched neatly using BOE.
2. Next, a layer of molybdenum was deposited between a SiNx layer and a SiO_2_ layer to prevent the metal structure from oxidizing. Using photolithography and RIE, the heating wires with varying line widths were patterned on the wafer, as shown in Figure 4d.
3. A thin composite membrane containing an ultrathin SiNx film and a layer of SiO_2_ was successively deposited via low-pressure chemical vapor deposition (LPCVD) on the wafer, fully covering the wafer. The ~100 nm thick SiO_2_ film was regarded as a coating layer to protect the transmission film from pollution and damage. Similar to the manufacturing process of transmission windows in the top chip, photolithography, RIE, and BOE were used to pattern and define the transmission window. After the transmission area was determined to be clean, the photoresist was stripped.
4. Contact pads were connected with the heating and measuring system on the sample rod. Electrodes were also patterned using photolithography, and the undesirable coated film was removed using BOE for about 3 min to open the pads. Care was taken when removing the photoresist so that the heated metal was easily oxidized.
5. The final photolithography was applied to define the gas holes and the suspended membrane. Then, the redundant composite film on which the gas holes and supporting membrane were located from previous processes on the back surface of the wafer was removed neatly by RIE. Next, three large windows were opened on the backside using RIE, as shown in Figure 4h. Lastly, the wafer protected by a fixture on the front side was wet-etched in KOH until the silicon nitride on the front side of the wafer.

**Figure 5:**
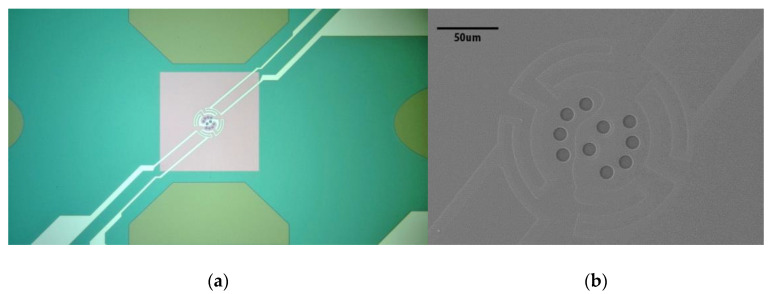
(**a**) Optical microscope photograph of the bottom chip; (**b**) SEM image of the bottom chip.

To clearly observe the partial details of the central structure, an optical microscope photograph and SEM image of the bottom chip are shown in Figure 5. As shown in Figure 5a, the transmission window was distributed in a winding heating element exactly located in the center of a large pink supporting membrane, and the four heating wires led from the center of the large supporting film constituted a pair of heating electrodes and a pair of measuring electrodes. As shown in Figure 5b, the distance and relative position of the heating element and the transmission window were quite consistent with the pre-designs.

## 4. Characteristics and Experimental Results

In order to further characterize the reliability of the microchip and the performance of the designed TEM chip structure, 20 microchips were selected and cleaned for testing after the wafer including many devices was cut using the laser scribing machine. Before the test of the microchips, the TEM, leak detection equipment, gas control system, and heating control system were established as being in good condition. In addition, samples of palladium nanoparticles were prepared.

### 4.1. Temperature Characteristic

Temperature uniformity is an important indicator of in situ TEM chips. Whether the temperature is uniform affects whether the reaction occurs synchronously. When the reaction occurs, the temperature of the material in the 10 observation windows should be as consistent as possible. To measure the temperature of 10 observation windows and, thus, obtain temperature uniformity, TCR and pyrometer measurements were used.

**Figure 6:**
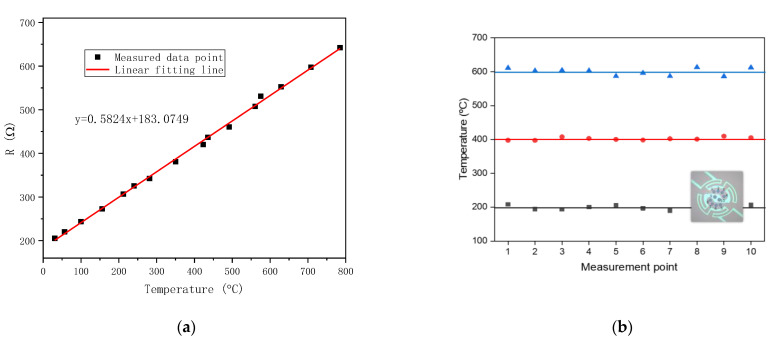
Temperature characteristics: (**a**) calibration of TCR; (**b**) temperature uniformity at 200 °C, 400 °C, and 600 °C of the 10 observation windows.

To evaluate the TCR of the bottom chip, the four-probe method was employed to measure the resistance of the metal structure. The temperature readings were obtained using an IR pyrometer. The obtained data points were plotted, and a linear fit was applied to derive the TCR, which was found to be 0.002913 °C^−1^ (Figure 6a). It was confirmed that the heater had a linear TCR across a wide temperature range, allowing precise temperature control through closed-loop feedback.

To assess the uniformity of temperature, measurements were taken at 10 observation windows around 200 °C, 400 °C, and 600 °C (Figure 6b). The results indicate that the temperature uniformity of all 10 observation windows exceeded 95.1% around 600 °C, temperature uniformity exceeded 96.8% around 400 °C, and temperature uniformity exceeded 95.5% around 200 °C.

### 4.2. Resolution Characteristic

Spatial resolution is one of the most vital performances of in situ TEM chips, reflecting the limit scale at which in situ TEM characterizes matter. Resolution profoundly affects whether we can capture the evolution of material structure and instantaneous changes in reactions. Combined with existing experimental equipment, we designed observation experiments to test spatial resolution.

**Figure 7:**
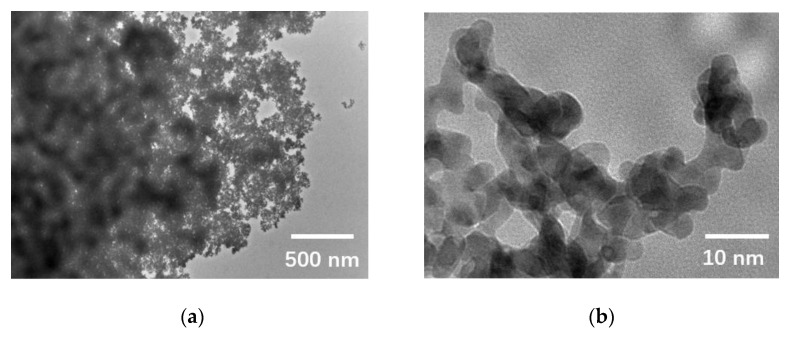

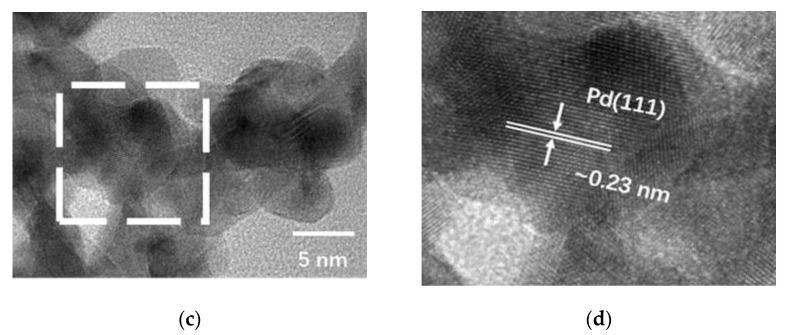
Resolution performance. Observation of palladium on a window using different scale bars: (**a**) 500 nm; (**b**) 10 nm; (**c**) 5 nm; (**d**) enlarged image of dotted box in Figure 7c shows the lattice reference spacing.

Prior to the assembly of the sample holder with the microchip, palladium nanoparticles were dispersed and coated on the bottom chip to facilitate the visualization of the sample particles through the observation window. The microchip’s tightness and gas pressure resistance were then assessed by placing the sample holder in a leak detection device. Subsequently, the microchip was inserted into the TEM cavity under high-vacuum conditions to ensure its structural integrity during analysis. Real-time observations of the palladium nanoparticles were conducted at room temperature under a flowing argon pressure of 1 bar (Figure 7). The results demonstrated clear visualization of the palladium lattice stripes on a 5 nm scale.

## 5. Conclusions

This paper presented a novel microchip that comprises a top and bottom chip, which was developed and used in conjunction with TEM for in situ observation. The microchip was designed in a way that ensures good temperature performance, and the positions of windows and metal wires were suitably defined. This research contributes to our understanding of the evolution of material structures and chemical processes in hot gas environments in real time. The microchip was tested and characterized in both atmospheric and high-vacuum environments. The microreactor features a very large observation window area of over 1130 μm^2^, achieving a temperature uniformity of over 95% in the electron transmission windows. In situ observations in a 1 bar flow atmosphere demonstrated lattice-level resolution. This wafer-level microchip with high resolution and outstanding temperature uniformity has enormous potential for explaining microscopic principles and mechanisms.

## Data Availability

The data presented in this study are available on request from the corresponding author.
